# Differential Expression Analysis in RNA-Seq by a Naive Bayes Classifier with Local Normalization

**DOI:** 10.1155/2015/789516

**Published:** 2015-08-03

**Authors:** Yongchao Dou, Xiaomei Guo, Lingling Yuan, David R. Holding, Chi Zhang

**Affiliations:** ^1^School of Biological Sciences, University of Nebraska, Lincoln, NE 68588, USA; ^2^Department of Agronomy and Horticulture, University of Nebraska, Lincoln, NE 68588, USA; ^3^Center for Plant Science and Innovation, University of Nebraska, Lincoln, NE 68588, USA

## Abstract

To improve the applicability of RNA-seq technology, a large number of RNA-seq data analysis methods and correction algorithms have been developed. Although these new methods and algorithms have steadily improved transcriptome analysis, greater prediction accuracy is needed to better guide experimental designs with computational results. In this study, a new tool for the identification of differentially expressed genes with RNA-seq data, named GExposer, was developed. This tool introduces a local normalization algorithm to reduce the bias of nonrandomly positioned read depth. The naive Bayes classifier is employed to integrate fold change, transcript length, and GC content to identify differentially expressed genes. Results on several independent tests show that GExposer has better performance than other methods. The combination of the local normalization algorithm and naive Bayes classifier with three attributes can achieve better results; both false positive rates and false negative rates are reduced. However, only a small portion of genes is affected by the local normalization and GC content correction.

## 1. Introduction

RNA-Seq is a technology based on next-generation sequencing to determine transcript abundance, transcriptional structure of genes, and posttranscriptional modifications. It is essential to accurately construct genome-wide gene expression profiles in order to interpret the functional elements of the genome, molecular constituents of cells, development of organisms, and mechanism of diseases [1]. RNA-seq has many advantages over microarray such as high resolution, low background noise, no requirement on prior knowledge of reference sequences, and the ability to distinguish isoforms and allelic expression [1]. RNA-seq data are typically generated from a library of cDNA fragments made from a population of mRNAs. Then cDNAs are sequenced* en masse* with or without amplification. There are two steps in analyzing the RNA-seq reads. The obtained short reads are first aligned to a reference genome or transcriptome, and, in the second step, for a given gene, the numbers of reads are compared between two different samples. The number of short reads mapped onto one gene is the count that is taken as a measure of the expression level of the gene. Many different types of analyses can be applied to the results of short-read alignment, including single nucleotide polymorphism discovery, alternative transcript identification, and gene expression profiling.

Because of the importance of RNA-seq, many methods have been developed to analyze aligned RNA-seq data to identify differentially expressed (DE) genes over the last four years. They include edgeR [2], DESeq [3], Cuffdiff [4], baySeq [5], TSPM [6], NBPSeq [7], BitSeq [8], POME [9], NOISeq [10], Gfold [11], and MRFSeq [12]. EdgeR [2], the first statistical method developed for digital gene expression data, is a parametric statistical method, which is based on a negative binomial model (an overdispersed Poisson model) [13]. DESeq [3] is also a parametric statistical method based on the negative binomial model. When estimating variances, DESeq and edgeR both employ gene information but edgeR estimates the gene-wise variance or dispersion by conditional maximum likelihood conditioning on the total count for that gene [14]. Cuffdiff [4], a part of the Cufflinks package developed for the identification of differentially expressed genes and revealing differential splicing events, uses a similar normalization method as DESeq and specifically addresses the uncertainties of read counts caused by ambiguous reads from different but similar isoforms. The baySeq [5], another parametric statistical method using a negative binomial model, takes a Bayesian approach which assumes that nondifferentially expressed genes should possess the same prior distribution on the underlying parameters across conditions, while differentially expressed genes should possess variant parameters for prior distributions. NBPSeq [7] is based on an overparameterized version of the negative binomial distribution that is called an NBP model. BitSeq [8] is a recently developed method, which estimates the distribution of transcript levels based on a probabilistic model of the read generation process and is simulated with a Markov chain Monte Carlo (MCMC) algorithm. BitSeq estimates the variance in the transcript expression based on a hierarchical log-normal model and determines the probability of differential expression by Bayesian model averaging. POME is another recently developed algorithm for gene expression analysis with RNA-seq, which uses Poisson mixed-effects model to characterize base-level read coverage within each transcript [9]. NOISeq [10] is a nonparametric statistical method, and several different normalization methods for the raw read counts are implemented with NOISeq, including RPKM (reads per kilobase of exon model per million mapped reads) [15], TMM [16], and UQUA [17]. Gfold is designed for samples without replicates, and significantly differentially expressed genes are determined based on the posterior distribution of their log fold changes [11]. MRFSeq [12] combines a Markov random field (MRF) model and the gene coexpression data to predict differential gene expression. Recently, a quantile normalization method has been developed to remove technical variability in RNA-seq data [18].

The transcript abundance of genes causes bias in detecting differential expression [19]. Nonuniform read coverage as a result of experimental protocols and bias caused by local sequence context also exists and some correction methods have been developed. The biases from the GC content can be corrected by base-level correction methods, such as the random hexamer bias correction method [20] and multiple additive regression trees (MART) [21]. Additional gene-level methods [22, 23] are developed to detect GC content biases and dinucleotide frequencies based on aggregated read counts for each gene and to remove the GC content bias trend across genes. Other types of GC content correction algorithms have also been developed [24, 25]. It has been reported that even after global normalization, longer transcripts are more likely to be called as differentially abundant compared to the shorter ones using *t*-tests [26]. Gao et al. developed an algorithm for transcript length normalization based on Poisson models; each gene's test statistics were adjusted using the square root of the transcript length followed by testing for gene set using the Wilcoxon rank-sum test [27]. Both positional [28] and sequence-specific [20, 29] biases are identified in sequenced fragments. Positional bias refers to a local effect in which fragments are preferentially located towards either the beginning or the end of transcripts, and sequence-specific bias is a global effect where the sequence surrounding the beginning or the end of the potential fragment affects its likelihood of being selected for sequencing. These biases can affect expression estimates [21], and Roberts et al. designed algorithms to correct these biases [23].

In our research, we have identified a new bias of mapped reads, the unevenly positioned reads depth, and designed a local normalization algorithm to correct this bias. Based on that, we have developed an analysis method using a naive Bayes (NB) classifier to determine DE genes. This method, called GExposer, uses three attributes: fold change (FC), averaged reads per kilobase (ARPK), and relative GC content (GCC). GExposer has performed the best or among the best when compared with other statistical methods and tested on multiple data sets beyond the one the model was constructed on. The software tool is available at http://sysbio.unl.edu/.

## 2. Materials and Methods

**Figure 1:**
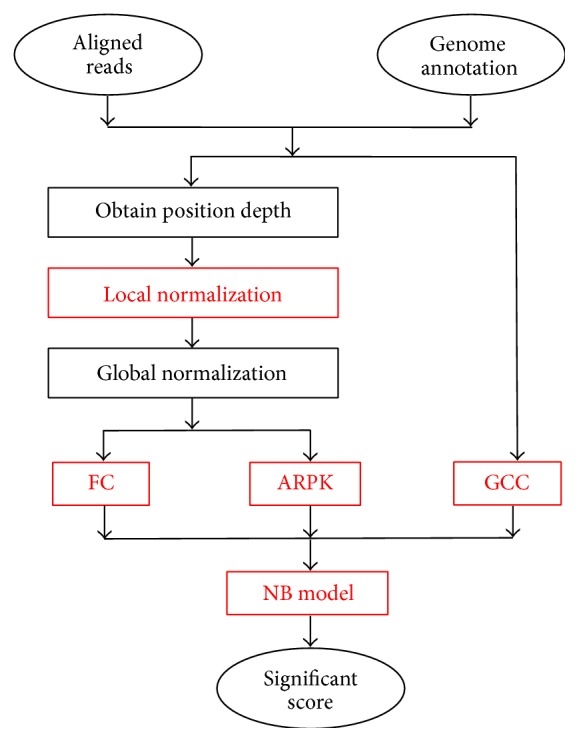
Flowchart of GExposer.

Figure 1 shows the flow chart of GExposer. The inputs of this tool are aligned reads and their genome annotations. All data were preprocessed with the same protocol. The raw short reads underwent the quality control with FASTX-Toolkit package [30], and all low quality reads, that is, average scores <20, were removed. Any short read mapping tool can be used to align RNA-seq reads to a reference genome. In this study, all RNA-seq reads were mapped by Bowtie [31] allowing up to two mismatches, and the reads mapped to multiple locations were discarded. Numbers of reads in genes were counted by the HTSeq-count tool using corresponding gene annotations, and the “union” resolution mode was used [32]. The distribution of read depths in each transcript can be determined according to the gene annotation as well. With a Poisson model, all read depths in one transcript are adjusted, and abnormal high or low depths are modified. This step is referred to as the local normalization. The local normalization can reduce the noise from nonspecific resources and the variation among replicates. Therefore, the variation among replicates is not considered as an attribute in the following NB classifier. Then, the global normalization algorithm, TMM, designed in DESeq package [3] is used to normalize reads depths between samples, and three attributes of the gene reads are extracted: FC, ARPK, and relative GC content. Generally, FC shows the relative difference between two samples and ARPK is the expression level of a transcript normalized by the transcript length. Previous studies also suggested that integrating GCC can help find DE genes [25], and, hence, GCC is included. Finally, a naive Bayes (NB) classifier is applied onto these features to score a given gene. The NB classifier is a simple probabilistic classifier based on applying Bayes theorem with naive independence assumptions. Generally, a probability model for a classifier is a conditional model *p*(*C*∣*f*_1_, *f*_2_,…, *f*_*n*_), where *C* is the class variable and *f*_*i*_ are attribute variables (e.g., GExposer has three attributes). Using Bayes' theorem, this conditional model can be transformed to

(1) $$\begin{array}{c} p\left(C\;|\;f_{1},f_{2},\ldots ,f_{n}\right)=\frac{p\left(C\right)p\left(f_{1},f_{2},\ldots ,f_{n}\;|\;C\right)}{p\left(f_{1},f_{2},\ldots ,f_{n}\right)}. \end{array}$$

Since a naive Bayes classifier has the conditional independence assumption, which assumes that, for a given class variable, the value of a particular attribute is unrelated to any other attributes, the conditional distribution over the class variable *C* becomes

(2) $$\begin{array}{c} p\left(C\;|\;f_{1},f_{2},\ldots ,f_{n}\right)=\frac{p\left(C\right)\prod_{i=1}^{n}p\left(f_{i}\;|\;C\right)}{p\left(f_{1},f_{2},\ldots ,f_{n}\right)}. \end{array}$$

All model parameters, including class priors and attribute probability distributions, can be estimated from the training set with the method of maximum likelihood. An advantage of an NB classifier is that it only requires a small amount of training data to estimate the parameters, such as means and variances of the variables. The software package used for this work is the *R* package, e1071, which computes the conditional a posteriori probabilities of a categorical class variable using the Bayes rule. For each gene, its feature vector has three values for the three attributes, which will be described in the following sections in detail. For the special requirement, we modified the calculation of FC, ARPK, and GCC from their common definitions in bioinformatics to a normalized version to make sure their ranges are in [0,1]. For the training step, the function of “naiveBayes” in the package e1071 is used to train a statistical model based on a training set, in which each gene has three attributes and known status. For the prediction step, the function of “prediction” can calculate the conditional a posteriori probability for a gene based on its feature vector with the three values. The conditional a posteriori probability is returned as a score, which is the probability of the given gene belonging to the class. The training data set and the *R* code are available for downloading from our website http://sysbio.unl.edu/.

### 2.1. Attributes

Three attributes are calculated with the following equations. The attribute of FC is calculated as

(3) $$\begin{array}{c} \text{FC}=\frac{1}{1+\left|\log_{2}\left(M_{1}/M_{2}\right)\right|}, \end{array}$$

where *M*_*i*_ (*i* = 1,2) are the mean values of the numbers of aligned reads for a given transcript in all replicates of sample *S*_*i*_ (*i* = 1,2). Here, *M*_*i*_ (*i* = 1,2) are values after both the local and global normalizations. The attribute of ARPK for a transcript is calculated as

(4) $$\begin{array}{c} \text{ARPK}=\frac{1}{1+\left|\text{lo}{\text{g}}_{2}\left(\left(1000\times \left(M_{1}+M_{2}\right)\right)/\left(2\times \text{TL}\right)\right)\right|}, \end{array}$$

where *M*_*i*_ (*i* = 1,2) have the same definition as in FC calculation and TL is the transcript length. The attribute of GCC can be calculated as

(5) $$\begin{array}{c} \text{GCC}=\frac{1}{1+c/C}, \end{array}$$

where *c* is the average GCC of reads mapped to a given transcript in both samples and *C* is the average GCC of all mapped reads. The GCC of one read is the ratio of the total number of guanine and cytosine to the length of the read. For each gene, the NB model will give a score in [0,1] by its three features. The higher the score, the higher possibility a gene is differentially expressed. Here, we also assume that the features of a given gene are independent of its specific biological background.

### 2.2. Local Normalization

Generally, reads are positioned randomly along every transcript in RNA-seq [33]. For a given transcript *T* with coordinates (*t*_1_, *t*_*n*_) on a chromosome, the number of reads starting from position *t*_*i*_ (*i* = 1,2,…, *n*) is defined as *r*_*i*_. The total number of reads mapped to the transcript is *R* = ∑*r*_*i*_. As the assumption that *r*_*i*_ is an accumulation of random events, *r*_*i*_ can be modeled as

(6) $$\begin{array}{c} r_{i}\sim p\left(\lambda_{T}\right), \end{array}$$

where *p*(*λ*_*T*_) is a Poisson distribution with parameter *λ*_*T*_. The unbiased estimate of *λ*_*T*_ is

(7) $$\begin{array}{c} \lambda_{T}=\frac{1}{n}\overset{n}{\underset{i=1}{\sum }}r_{i}. \end{array}$$

Confidence limits are the lower and upper boundaries of a confidence interval. With the Poisson distribution, we can find the upper confidence limits (UCL) and lower confidence limits (LCL) for *λ*_*T*_, such as at 97.5% and 2.5% confidence level, respectively, in this paper. With the UCL and LCL, the number of reads for a given position, *r*_*i*_, can be corrected as

(8) $$\begin{array}{c} r_{i}^{0}=\left\{\begin{array}{cc} \text{UCL}, & \text{if}\;r_{i}>\text{UCL},\\ \text{LCL}, & \text{if}\;r_{i}<\text{LCL},\\ r_{i}, & \text{othewise}. \end{array}\right. \end{array}$$

With this adjustment, the total number of reads mapped to the transcript is

(9) $$\begin{array}{c} R^{0}=\overset{n}{\underset{i=1}{\sum }}r_{i}^{0}. \end{array}$$

It has been reported that the assumption of a Poisson distribution is too restrictive to predict more accurate variations among data from different replicates [2, 3], but a Poisson distribution can fit data in a specific exon from one sample, because they have fewer variations.

### 2.3. Training and Testing Data Set

We collected training and testing data sets from several different resources, and Table 1 summarized these data sets. The training data set contains two RNA-seq data sets with 35 base-pair-long reads obtained using Illumina's Genome Analyzer II high-throughput sequencing system [17], and they correspond to data obtained by the microarray quality control (MAQC) project [34]. The accession number of these RNA-seq data in SRA is SRA010153.1. The two RNA sample types used were a universal human reference RNA (UHRR) from Stratagene and a human brain reference RNA (HBRR) from Ambion. There are seven lanes for each sample with about 40 million reads. After processing the RNA-seq data, all RNA-seq reads were aligned against the human genome (GRCh37.68). The data set has about 997 RT-PCR data for validation of RNA-seq analysis results [35], and the genes with mean reads number fewer than 5 in both samples are not considered. Based on their expression (log_2_ fold change), the genes were grouped into three sets: DE, no-call, and non-DE (NDE), with the log_2_(fold-change) being >1.5 [0.5, 1.5] and <0.5, respectively. The expression log_2_(fold-change) for RT-PCR samples was calculated by the ΔΔCT method [36]. This way, we compiled 389, 178, and 235 genes in the categories of DE, no-call, and NDE, respectively. For the same RNA-seq data, corresponding microarray experiments were conducted by MAQC with Affymetrix Human Genome U133 Plus 2.0 arrays (GEO: GSE5350). Microarray data were preprocessed with RMA [37] and analyzed with limma package [38]. For the results of the microarray data analysis, genes having absolute log_2_(fold-change) ≥1.5 and *P* values <10^−3^ were considered as DE; genes were NDE if their absolute log_2_(fold-change) <0.5, and the rest were no-call genes. Finally, there are 1756, 2340, and 3372 genes for DE, no-call, NDE, respectively. The classified genes by both PCR and microarray were combined together to be used as the training data set for the NB model. A gene was considered as DE (or NDE) if at least one method, either PCR or microarray assay, confirmed it as DE (or NDE), and finally there are 1966 DE and 3388 NDE genes in this training set. The NB model trained by this data set is also applied to other species, including plants, for testing. More details about other test data sets are described in the following sections.

## 3. Results and Discussion

### 3.1. Results for the Training Data Set

**Figure 2:**
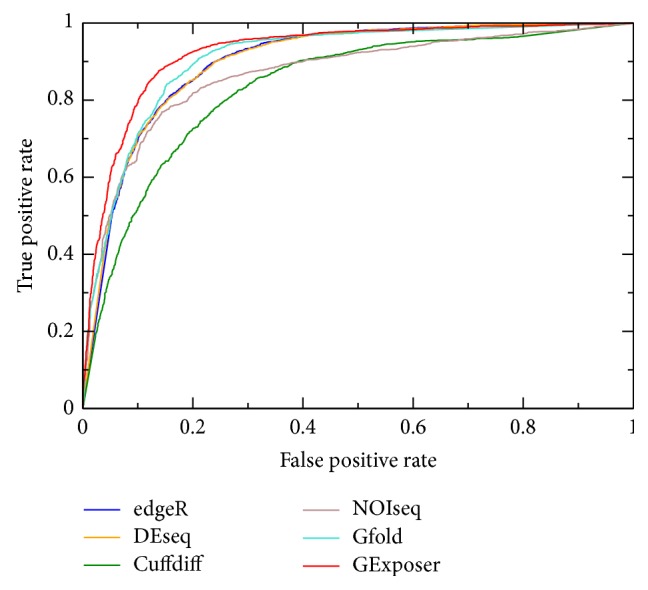
ROC curves of different methods tested on the training data set.

The NB model was trained using 1966 DE and 3388 NDE genes in the training data set. DE genes were considered as positive while NDE ones were considered as negative. For validation, the leave-one-out cross-validation was used to score them. To assess the performance of GExposer, the current most popular methods such as edgeR (2.6.7) [2], DESeq (1.12.0) [3], Cuffdiff (2.1.1) [4], NOISeq (2.0) [10], and Gfold (1.0.7) [11] were applied to the same RNA-seq data sets for comparison. The default setups were used for other methods as well. Following the work of Tarazona et al. [10], *P* values created by the other methods, except for NOIseq, were used as the scores for ranking genes. NOIseq outputs one score for each gene to quantify the expression level. Since different parameters are used by different methods to select DE genes, it is difficult to select a cutoff that can produce comparable analysis and fair comparison for all methods. In this study, we compared the area under receiver operating characteristic curve (AUC) values of all methods, which can avoid the difficulty of selecting a comparable cutoff of *P* values for all methods. This evaluation method has been used to other RNA-seq data analysis tools before [17, 39]. A receiver operating characteristic (ROC) curve represents a dependency of sensitivity and (1 − specificity), which is plotted with true positives rate versus false positive rate at various threshold settings. To change the threshold setting, the number of the predicted DE genes was increased in steps of one gene.  Figure 2 shows ROC curves of all methods for the same data set. The AUC values of all methods are shown in Table 2. GExposer achieved the highest AUC value (0.9255). To test the ability of each method to successfully identify DE (true positive) or NDE (true negative) from a noisy pool, no-call genes are treated as true negative (NDE) or true positive (DE) genes, respectively. For no-call genes, the model trained by all DE and NDE genes was used to score them with an NB classifier. All AUC values are also shown in Table 2. For both cases, GExposer achieved the highest AUC values.

### 3.2. Independence to the Number of Replicates

To study the dependence on the number of replicates, six RNA-seq analysis methods were applied on some subsets of the training data set, from one lane to seven lanes. Results of these methods are shown in Table 3. Results of all methods are relatively stable with more than one lane, but there is a sharp drop in AUC values for Cuffdiff and DESeq when only one lane is available. If there is no replicate, the AUC value for DESeq is no more than 0.67 and for Cuffdiff is no more than 0.60. GExposer constantly has the largest AUC values for different numbers of replicates. Moreover, although Gfold is designed for data without replicate, GExposer still outperformed it even when only using one lane. This test implies that the local normalization reduces the variation among replicates, and, hence, the performance of GExposer is not affected by replicates.

### 3.3. Results from Human Colorectal Cancer Data Set

An RNA-seq data set for human colorectal cancer generated by Griffith et al. [40] was used to perform an independent test. This data set is from the same species as the training set, but from a different tissue. In this data set, 84 bp paired-end reads were sequenced, and there are eight lanes in total for colorectal cancer cell line MIP/5FU and 15 lanes for cell line MIP101 [40]. In this data set, the top 50 differential or alternative expression events were tested and 13 genes were confirmed as DE by experiments. For the human colorectal cancer data set, all 23 lanes with 84 bp length reads were applied to different RNA-seq analysis tools, and the 13 known DE genes were ranked using these tools. The orders of these genes ranked by different methods are shown in Table 4. One can find that 7 out of the 13 confirmed genes are ranked in the top 50 genes by GExposer. The numbers for other methods are no more than six except for Gfold. In particular, Gene TSPAN12 is ranked in top 50 only by GExposer and Cuffdiff. In this data set, some genes, such as MR1, have long transcript length with many exons, and an isoform with a small portion of exons is expressed. GExposer could not rank this kind of genes to top position because of the transcript length correction. Using the length of a specific isoform, instead of the total length of all exons for a given gene, could be considered to improve GExposer's accuracy of identification of differentially expressed isoform.

### 3.4. Performance of GExposer on Maize RNA-Seq

In contrast to other RNA-seq analysis tools, GExposer needs a training set, which was a set of RNA-seq data from human tissues for this work. Naturally, one may raise the question whether the performance of GExposer could have potential correlation with the species that is used to generate the RNA-seq data. To test whether the human-data trained GExposer works well on different species, the method was applied to plant (maize) RNA-seq data. The maize RNA-seq data set that compares bundle sheath and mesophyll cells were obtained from the laser-capture microdissected (LCM) samples from the tip of the maize leaf which incorporates two biological replicates [41]. The RNA-seq data were obtained from the NCBI short read archive (SRA) under accession number SRA012297. The expression levels of 40 genes (only 37 genes were found in the current version of B73 gene annotation) were measured by RT-PCR and were also grouped into three sets: DE (6), no-call (22), and NDE (9), as log_2_(fold-change) is >1.5 [0.5, 1.5] and <0.5, respectively, for the comparison between cells at a maturing zone (+4 cm above the leaf two ligule) and mature zone (tip, +1 cm below the leaf three tip). All RNA-seq analysis tools were applied onto this RNA-seq data set, and these 37 genes were ranked by different methods. The distributions of top 10 genes ranked by different methods are shown in Table 5. In top 10 genes, GExposer has the largest number of DE genes (5 genes), while DESeq has the least (3 genes). None of these methods rank NDE genes in top 10, except for Gfold.

### 3.5. Performance of Local Normalization

**Figure 3:**
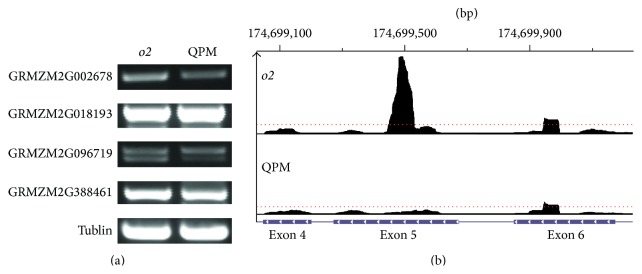
(a) RT-PCR results of four genes in* o2* and QPM lines. (b) Short read distribution in three exons of GRMZM2G002678 in W64* o2* and QPM, and the red dotted line indicates the adjusted depth by local normalization method.

GExposer was also applied onto the maize RNA-seq data to test the performance of the local normalization method. The RNA-seq data were generated to study differentially expressed genes in quality protein maize (QPM) endosperm tissue [42]. To simplify the results, the RNA-seq data from the two genotypes, W64A* o2* mutant and K0326Y QPM, were used because it is the most important comparison for the QPM study. Each sample has about 20 million 50 bp long reads made up of kernels pooled from five biological replicates. Reads for maize were aligned against the reference genome (ZmB73-RefgenV2), for the pair-wise comparison between the genotypes, W64* o2* and K0326Y QPM. There is a portion of genes that are assigned with different expression levels when using or not using the local normalization method. We selected seven such genes that have been assigned different fold changes with and without local normalization and applied RT-PCR experiments to validate the real fold change. Since the gene annotations and sequences of B73 are used to design the primers for these genes in W64* o2* and K0326Y QPM, only four genes, GRMZM2G002678, GRMZM2G018193, GRMZM2G096719, and GRMZM2G38846, showed results for RT-PCR experiments. The details of the RT-PCR experiment and primers of these four genes are described in Supplementary Data (see Supplementary Material available online at http://dx.doi.org/10.1155/2015/789516). For these four genes, their log_2_(fold-change) measured using the local normalization method and not using it is shown in Table 6. The log_2_(fold-change) measured without the local normalization step shows higher values, while that measured by GExposer with the local normalization is no more than 0.61. The RT-PCR results support the GExposer analysis with the local normalization. The RT-PCR results of these four genes are shown in Figure 3(a), and these four genes are not differentially expressed in W64* o2* and K0326Y QPM. To understand what causes the difference, read coverage of three exons of GRMZM2G002678 are shown in Figure 3(b). Many reads aggregate in a very narrow peak in exon 5 for W64* o2*, but there is no peak in K0326Y QPM. Therefore, this peak artificially inflates the absolute value of fold changes between W64* o2* and K0326Y QPM. With the use of the local normalization method, the adjusted depth is low (at the level of the red dotted line), and this false positive is therefore removed.

### 3.6. Difference between True and Simulated Data Sets

**Figure 4:**
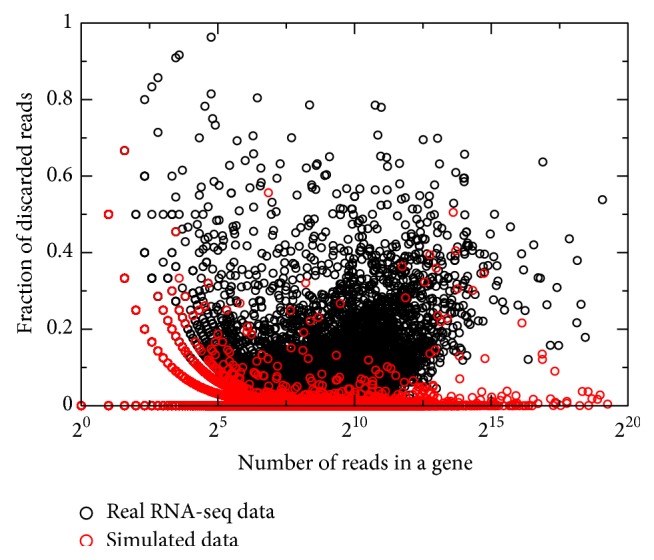
Fractions of discarded reads by local normalization method for both real and simulated RNA-seq data.

In order to further evaluate the effect of nonrandomly positioned reads, the real and simulated RNA-seq data sets were compared. The next-generation sequencing read simulator “ART” [43] was used to simulate RNA-seq reads. To simulate the sequencing, the sequencing read simulator assumes that the reads uniformly and randomly distribute on the transcript [33], and, hence, there is no abnormal peak to be adjusted by the local normalization method. We randomly selected W64* o2* RNA-seq data from the maize endosperm data set as the template to conduct the simulation. For each transcript, the same number of reads as in the template data set was generated by the simulator. Then, all simulated reads were mapped to the same genome with the same parameters as for the W64* o2* RNA-seq data. The fractions of the discarded reads by the local normalization method of each transcript are shown in Figure 4, and there is a significant difference between the real and simulated data sets. For the simulated data, only several genes (0.8% of all genes) have more than 10% reads discarded with the local normalization, but thousands of genes (10.3%) for the real RNA-seq data do. It indicates that in reality, sometimes, reads are not uniformly, randomly sequenced on a transcript, and the local normalized method is a necessary step for this kind of cases.

### 3.7. Assessment of Each Attribute in GExposer

GExposer used three attributes: FC, ARPK, and relative GCC. To understand which one in the three scores plays a more important role, each attribute was removed from the system and the same training and test procedures were conducted to the training data set. The results are shown in Table 7. The absence of any attribute leads to some decrease of the AUC value, but the attribute of FC is the most significant. The largest changes occurred when the FC attribute was removed, whereas removing ARPK and GCC only caused small changes of AUC (0.0464 and 0.0227). It is not surprising that the fold change of read numbers is the major criterion to determine DE genes. The attribute ARPK is related to the expression level of a given gene and, hence, also plays an important role in the DE gene identification. The GCC correction is applied to a very small portion of genes. Therefore, the absence of the correction reduces the AUC value only slightly, although this correction is important for those specific genes.

### 3.8. Local Normalization and GC Content

**Figure 5:**
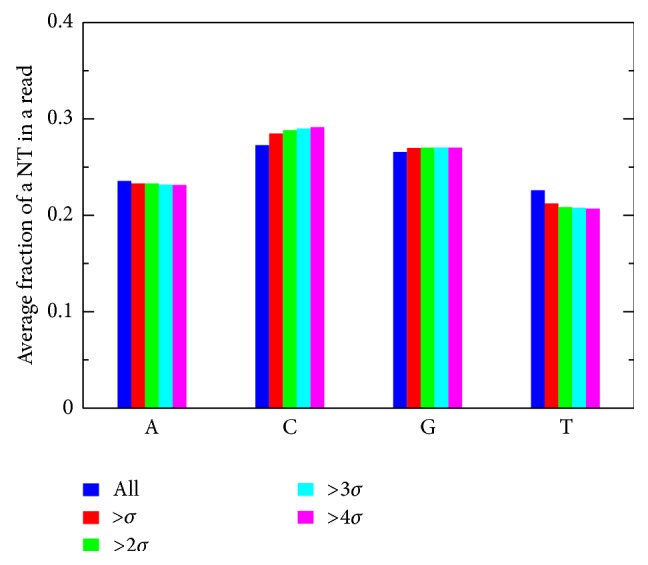
The distributions of nucleotides with different depths.

The local normalization method mainly focuses on the high peaks of the reads; for a given high peak, its depth will be modified according to the average depth and its standard deviation. This raises one question: do the reads in these peaks have special patterns of nucleotides or GCCs, compared with the sequence background of all reads? If they have a special GCC, for example, some existing correction algorithms for GCC bias [20–23] could be applied to this case, instead of using the local normalization. To answer this question, we calculated the distributions of nucleotides in different types of reads. The result on maize W64* o2* data set from the QPM RNA-seq data is shown in Figure 5. The blue bars are for all aligned reads, and the red, yellow, green, and brown bars are for the reads from peaks with depths of 1, 2, 3, and 4 standard deviations away from the average. The average portion of a type of nucleotide (NT) in one read was calculated. From Figure 5, one can see that, for a certain type of NT, different types of depths have very similar portions. This indicates that the abnormal high peaks of reads have no correlation with the GCC. Therefore, we can conclude that this kind of read abundance does not result from a special pattern of nucleotides.

## 4. Conclusions

In the study, a new bias in RNA-seq data called nonrandomly positioned reads was identified. Our analysis shows that this bias is different from GCC bias. In order to reduce the bias, a local normalization algorithm has been developed and the false positive rate caused by this bias is reduced, which has been validated by the RT-PCR experiments. Moreover, the combination of three attributes, FC, ARPK, and GCC, can achieve better results; both false positive rates and false negative rates are reduced. However, GCC correction is only applied to a very small portion of genes in a whole genome. The model of GExposer was trained by one data set, and there is great potential for machine learning methods to improve the performance in finding DE genes by combining more training data sets from different species. On the other hand, training data from various species could potentially limit the ability of a naive Bayes classifier to identify DE genes.

## Supplementary Material

In the Supplementary Material, RT-PCR method used to validate gene expression in maize is described, and the primer sequences used for four maize genes are displayed.

## Figures and Tables

**Table 1:** Summary of all data sets used in this paper.

Data set	DE genes	NDE genes	SRA accession number
MAQC UHRR and HBRR	1966	3388	SRA010153.1
Colorectal cancer	13	0	SRX026158 and SRX026158
Maize leaf	6	9	SRA012297

**Table 2:** AUC values of six methods on the training data set with the leave-one-out cross-validation.

Method		False positive test No-call as NDE	False negative test No-call as DE
edgeR	0.8997	0.8567	0.7945
DESeq	0.9002	0.8602	0.7909
Cuffdiff	0.8347	0.7740	0.7610
NOISeq	0.8679	0.8460	0.7267
Gfold	0.9079	0.8886	0.7790
GExposer	0.9255	0.9030	0.8054

**Table 3:** AUC values of six methods on the training data set with different number of replicates.

Method	1	2	3	4	5	6	7
False positive test, no-call as NDE							
edgeR	0.8535	0.8607	0.8595	0.8591	0.8584	0.8574	0.8567
DESeq	0.6621	0.8626	0.8622	0.8618	0.8613	0.8610	0.8602
Cuffdiff	0.5963	0.7904	0.7772	0.7904	0.7773	0.7748	0.7740
NOIseq	0.8334	0.8392	0.8425	0.8445	0.8452	0.8456	0.8460
Gfold	0.8870	0.8334	0.8871	0.8875	0.8874	0.8886	0.8886
GExposer	0.8968	0.9016	0.9024	0.9024	0.9028	0.9032	0.9030

False negative test, no-call as DE							
edgeR	0.7845	0.7903	0.793	0.7934	0.7936	0.7934	0.7945
DESeq	0.5800	0.7871	0.7895	0.7902	0.7912	0.7905	0.7909
Cuffdiff	0.5894	0.7674	0.7645	0.7674	0.7623	0.7606	0.7610
NOIseq	0.7269	0.7342	0.7335	0.7308	0.7288	0.7276	0.7267
Gfold	0.7905	0.6702	0.7498	0.7670	0.7753	0.7753	0.7790
GExposer	0.7942	0.8015	0.8045	0.8051	0.8053	0.8051	0.8054

**Table 4:** Ranking of 13 genes by six different methods.

Gene	edgeR	DESeq	cuffdiff	NOIseq	Gfold	GExposer
LAPTM4B	109	109	99	**11**	**27**	**8**
TSPAN12	59	54	18	55	98	**10**
TNNI2	8750	8730	41066	671	241	15861
H19	**7**	**6**	**2**	**1**	**2**	**25**
ZNF185	651	672	56286	7879	81	1008
MR1	144	141	22837	51	**12**	4156
ASRGL1	125	269	8560	101	**10**	854
C12orf59	**13**	**11**	70	**4**	**1**	**2**
KLK6	680	646	1568	**17**	**36**	115
ATOH8	66	70	956	224	**21**	99
FUT3	57	62	1583	**6**	**5**	**9**
KRT20	356	625	462	399	**33**	**18**
OLR1	**48**	**42**	8741	**7**	**4**	**4**

The numbers in bold font correspond to genes that were ranked in top 50.

**Table 5:** The distributions of top 10 differentially expressed genes ranked by six different methods on maize RNA-seq data.

	DE	No-call	NDE
edgeR	4	6	0
DESeq	3	7	0
Cuffdiff	4	6	0
NOIseq	4	6	0
Gfold	4	5	1
GExposer	5	5	0

**Table 6:** Results of four maize genes with and without the local normalization.

Method	GRMZM2 G002678	GRMZM2 G018193	GRMZM2 G096719	GRMZM2 G388461
log_2_(FC) without local normalization	−1.20	1.69	−1.72	4.18
log_2_(FC) with local normalization	−0.61	0.40	−0.59	0.43

**Table 7:** Performance of GExposer omitting each attribute.

		False positive test No-call as NDE	False negative test No-call as DE
ΔGCC	0.9028	0.8964	0.8017
ΔARPK	0.8791	0.8656	0.747
ΔFC	0.6315	0.5946	0.6141
GExposer	0.9255	0.903	0.8054

## References

[1] Wang Z., Gerstein M., Snyder M. (2009). RNA-Seq: a revolutionary tool for transcriptomics. *Nature Reviews Genetics*.

[2] Robinson M. D., McCarthy D. J., Smyth G. K. (2010). edgeR: a bioconductor package for differential expression analysis of digital gene expression data. *Bioinformatics*.

[3] Anders S., Huber W. (2010). Differential expression analysis for sequence count data. *Genome Biology*.

[4] Trapnell C., Williams B. A., Pertea G. (2010). Transcript assembly and quantification by RNA-Seq reveals unannotated transcripts and isoform switching during cell differentiation. *Nature Biotechnology*.

[5] Hardcastle T. J., Kelly K. A. (2010). baySeq: empirical Bayesian methods for identifying differential expression in sequence count data. *BMC Bioinformatics*.

[6] Auer P. L., Doerge R. W. (2011). A two-stage Poisson model for testing RNA-Seq data. *Statistical Applications in Genetics and Molecular Biology*.

[7] Di Y. M., Schafer D. W., Cumbie J. S., Chang J. H. (2011). The NBP negative binomial model for assessing differential gene expression from RNA-Seq. *Statistical Applications in Genetics and Molecular Biology*.

[8] Glaus P., Honkela A., Rattray M. (2012). Identifying differentially expressed transcripts from RNA-seq data with biological variation. *Bioinformatics*.

[9] Hu M., Zhu Y., Taylor J. M. G., Liu J. S., Qin Z. S. (2012). Using poisson mixed-effects model to quantify transcript-level gene expression in RNA-Seq. *Bioinformatics*.

[10] Tarazona S., García-Alcalde F., Dopazo J., Ferrer A., Conesa A. (2011). Differential expression in RNA-seq: a matter of depth. *Genome Research*.

[11] Feng J., Meyer C. A., Wang Q., Liu J. S., Liu X. S., Zhang Y. (2012). GFOLD: a generalized fold change for ranking differentially expressed genes from RNA-seq data. *Bioinformatics*.

[12] Yang E.-W., Girke T., Jiang T. (2013). Differential gene expression analysis using coexpression and RNA-Seq data. *Bioinformatics*.

[13] Robinson M. D., Smyth G. K. (2008). Small-sample estimation of negative binomial dispersion, with applications to SAGE data. *Biostatistics*.

[14] Smyth G. K., Verbyla A. P. (1996). A conditional likelihood approach to residual maximum likelihood estimation in generalized linear models. *Journal of the Royal Statistical Society. Series B. Methodological*.

[15] Mortazavi A., Williams B. A., McCue K., Schaeffer L., Wold B. (2008). Mapping and quantifying mammalian transcriptomes by RNA-Seq. *Nature Methods*.

[16] Robinson M. D., Oshlack A. (2010). A scaling normalization method for differential expression analysis of RNA-seq data. *Genome Biology*.

[17] Bullard J. H., Purdom E., Hansen K. D., Dudoit S. (2010). Evaluation of statistical methods for normalization and differential expression in mRNA-Seq experiments. *BMC Bioinformatics*.

[18] Hansen K. D., Irizarry R. A., Wu Z. (2012). Removing technical variability in RNA-seq data using conditional quantile normalization. *Biostatistics*.

[19] Wu Z., Jenkins B. D., Rynearson T. A. (2010). Empirical bayes analysis of sequencing-based transcriptional profiling without replicates. *BMC Bioinformatics*.

[20] Hansen K. D., Brenner S. E., Dudoit S. (2010). Biases in Illumina transcriptome sequencing caused by random hexamer priming. *Nucleic Acids Research*.

[21] Li J., Jiang H., Wong W. H. (2010). Modeling non-uniformity in short-read rates in RNA-Seq data. *Genome Biology*.

[22] Zheng W., Chung L. M., Zhao H. (2011). Bias detection and correction in RNA-Sequencing data. *BMC Bioinformatics*.

[23] Roberts A., Trapnell C., Donaghey J., Rinn J. L., Pachter L. (2011). Improving RNA-Seq expression estimates by correcting for fragment bias. *Genome Biology*.

[24] Benjamini Y., Speed T. P. (2012). Summarizing and correcting the GC content bias in high-throughput sequencing. *Nucleic Acids Research*.

[25] Risso D., Schwartz K., Sherlock G., Dudoit S. (2011). GC-content normalization for RNA-Seq data. *BMC Bioinformatics*.

[26] Oshlack A., Wakefield M. J. (2009). Transcript length bias in RNA-seq data confounds systems biology. *Biology Direct*.

[27] Gao L., Fang Z., Zhang K., Zhi D., Cui X. (2011). Length bias correction for RNA-seq data in gene set analyses. *Bioinformatics*.

[28] Bohnert R., Rätsch G. (2010). rQuant.web: a tool for RNA-Seq-based transcript quantitation. *Nucleic Acids Research*.

[29] Srivastava S., Chen L. (2010). A two-parameter generalized Poisson model to improve the analysis of RNA-seq data. *Nucleic Acids Research*.

[30] Taylor J., Schenck I., Blankenberg D., Nekrutenko A. (2007). Using galaxy to perform large-scale interactive data analyses. *Current Protocols in Bioinformatics*.

[31] Langmead B., Trapnell C., Pop M., Salzberg S. L. (2009). Ultrafast and memory-efficient alignment of short DNA sequences to the human genome. *Genome Biology*.

[32] Anders S., Pyl P. T., Huber W. (2015). HTSeq—a Python framework to work with high-throughput sequencing data. *Bioinformatics*.

[33] Richard H., Schulz M. H., Sultan M. (2010). Prediction of alternative isoforms from exon expression levels in RNA-Seq experiments. *Nucleic Acids Research*.

[34] Shi L., Reid L. H., Jones W. D. (2006). The MicroArray Quality Control (MAQC) project shows inter- and intraplatform reproducibility of gene expression measurements. *Nature Biotechnology*.

[35] Canales R. D., Luo Y., Willey J. C. (2006). Evaluation of DNA microarray results with quantitative gene expression platforms. *Nature Biotechnology*.

[36] Benjamini Y., Hochberg Y. (1995). Controlling the false discovery rate—a practical and powerful approach to multiple testing. *Journal of the Royal Statistical Society Series B: Methodological*.

[37] Irizarry R. A., Hobbs B., Collin F. (2003). Exploration, normalization, and summaries of high density oligonucleotide array probe level data. *Biostatistics*.

[38] Smyth G. K. (2004). Linear models and empirical Bayes methods for assessing differential expression in microarray experiments. *Statistical Applications in Genetics and Molecular Biology*.

[39] Soneson C., Delorenzi M. (2013). A comparison of methods for differential expression analysis of RNA-seq data. *BMC Bioinformatics*.

[40] Griffith M., Griffith O. L., Mwenifumbo J. (2010). Alternative expression analysis by RNA sequencing. *Nature Methods*.

[41] Li P., Ponnala L., Gandotra N. (2010). The developmental dynamics of the maize leaf transcriptome. *Nature Genetics*.

[42] Guo X., Ronhovde K., Yuan L. (2012). Pyrophosphate-dependent fructose-6-phosphate 1-phosphotransferase induction and attenuation of Hsp gene expression during endosperm modification in quality Protein Maize. *Plant Physiology*.

[43] Huang W., Li L., Myers J. R., Marth G. T. (2012). ART: a next-generation sequencing read simulator. *Bioinformatics*.

