# Supplementary material for: Differential Expression Analysis in RNA-Seq by a Naive Bayes Classifier with Local Normalization
Source: Biomed Res Int. 2015 Aug 3;2015:789516. doi: 10.1155/2015/789516 (PMC4538581; doi:10.1155/2015/789516)
Supplement: Supplementary file 1 — In the Supplementary Material, RT-PCR method used to validate gene expression in maize is described, and the primer sequences used for four maize genes are displayed. [file 789516.f1.pdf]

RT-PCR

For RT-PCR verification of gene expression in maize, total RNA was extracted from endosperms of W64 *o*2 and K0326Y QPM. Total RNA was DNase 1 treated and then purified using Qiagen Rneasy cleanup kit. The cDNA was synthesized from 2 μg samples of cleaned RNA using IScript plus (Bio-Rad, Hercules, CA), according to the manufacturer's instructions. The cDNA was diluted five fold in water and subjected to regular RT-PCR with primers designed.

Primers used for RT-PCR:

GRMZM2G002678
F: 5'-TGTCATCAGCACAAGGGTGT-3'
R: 5'-CGAGCGTGAATCCTAAGAGG-3'

GRMZM2G018193
F: 5'-CTCAGCAACAATAGTTTCTTCCA-3'
R: 5'-TATTTCTGGGAAGCCACAAACAT-3'

GRMZM2G096719
F: 5'-CCTCCTCTCTCAATTCTTGCAG-3'
R: 5'-ATAACAAGGAGGGAAAAACATGAA-3'

GRMZM2G388461
F: 5'-TTATTCCACAATGCTCACTTGC-3'
R: 5'-AGCAGTTGTTGCTGTTGTAGGTAG-3'

Tubulin
F: 5'-GTGTCCTGTCCACCCACTCTCT-3'
R: 5'-GGAACTCGTTCACATCAACGTTC-3'
